# Physiological Mineralization during In Vitro Osteogenesis in a Biomimetic Spheroid Culture Model

**DOI:** 10.3390/cells11172702

**Published:** 2022-08-30

**Authors:** Maximilian Koblenzer, Marek Weiler, Athanassios Fragoulis, Stephan Rütten, Thomas Pufe, Holger Jahr

**Affiliations:** 1 Department of Anatomy and Cell Biology, Uniklinik RWTH Aachen, 52074 Aachen, Germany; 2 Institute for Experimental Molecular Imaging, RWTH Aachen University, 52074 Aachen, Germany; 3 Electron Microscope Facility, Uniklinik RWTH Aachen, 52074 Aachen, Germany; 4 Department of Orthopaedic Surgery, Laboratory for Experimental Orthopedics, Maastricht University Medical Centre, 6229 ER Maastricht, The Netherlands

**Keywords:** spheroid culture, osteoblast progenitors, MC3T3-E1, osteogenic differentiation, physiological biomineralization, volumetric micro-CT quantification, Fourier-transform infrared spectroscopy (FT-IR)

## Abstract

Bone health-targeting drug development strategies still largely rely on inferior 2D in vitro screenings. We aimed at developing a scaffold-free progenitor cell-based 3D biomineralization model for more physiological high-throughput screenings. MC3T3-E1 pre-osteoblasts were cultured in α-MEM with 10% FCS, at 37 °C and 5% CO_2_ for up to 28 days, in non-adherent V-shaped plates to form uniformly sized 3D spheroids. Osteogenic differentiation was induced by 10 mM β-glycerophosphate and 50 µg/mL ascorbic acid. Mineralization stages were assessed through studying expression of marker genes, alkaline phosphatase activity, and calcium deposition by histochemistry. Mineralization quality was evaluated by Fourier transformed infrared (FTIR) and scanning electron microscopic (SEM) analyses and quantified by micro-CT analyses. Expression profiles of selected early- and late-stage osteoblast differentiation markers indicated a well-developed 3D biomineralization process with strongly upregulated *Col1a1*, *Bglap* and *Alpl* mRNA levels and type I collagen- and osteocalcin-positive immunohistochemistry (IHC). A dynamic biomineralization process with increasing mineral densities was observed during the second half of the culture period. SEM–Energy-Dispersive X-ray analyses (EDX) and FTIR ultimately confirmed a native bone-like hydroxyapatite mineral deposition ex vivo. We thus established a robust and versatile biomimetic, and high-throughput compatible, cost-efficient spheroid culture model with a native bone-like mineralization for improved pharmacological ex vivo screenings.

## 1. Introduction

Bone fractures are the most common large-organ, traumatic injuries to humans and about 10% of fractures will not heal normally [1]. The canonical Wnt (cWnt) signaling pathway plays an essential role in the regulation of bone homeostasis [2]. Its dysregulation contributes to various bone phenotypes [3], ranging from osteoporosis (OP) to high bone mineral density (BMD) disorders, like osteopetrosis [4], often involving Wnt ligands or Wnt co-receptors [5].

Our demographic transition will further lead to a steady increase in age-related skeletal diseases like OP, the treatment of which remains a major clinical challenge. OP is characterized by low bone mass, due to a misbalance between bone formation and bone resorption, and is associated with an increased risk of fracture [6]. Currently, every second woman suffers at least one postmenopausal bone fracture [7,8], while current therapies are mainly based on pharmacological inhibitors of bone resorption [3]. More recent osteoanabolic strategies [4] focus on stimulating bone formation by targeting osteoblast cell lineage [9]. The development of such anabolic drugs requires cell-based assays that provide valid data on pharmacological efficacies of potential lead compounds. To this end, lithium has clinically been used safely and effectively for over half a century [10]. One of the well-characterized cell biologic actions of this cell membrane permeable ion is the inhibition of glycogen synthase kinase-3β, which consequently activates canonical Wnt (cWnt) signaling to Lrp5-independently increase bone formation and bone mass in mice [11].

Surprisingly, research on osteoblast lineage cells still largely relies on highly simplified 2D in vitro models. Obviously, monolayer cultures on hard plastic or glass surfaces discourage the formation of three-dimensional interactions and thus fail to appropriately mimic osteoblastic differentiation in vivo [12]. Hence, in bone research there is an urgent need for novel in vitro assays that better mimic this three-dimensional tissue architecture and allow for studying the effects of pharmacological intervention on osteoblast differentiation under biomimetic conditions [12].

Spheroid culture systems are a proven strategy to overcome the highly limited and bias-prone 2D approach. As compared to other techniques, such as biogel- or scaffold-based 3D culture models, the huge advantages of assumption- and support matrix-free gravitation-based spheroid culture systems are simplicity, cost-effectiveness, and suitability for high-throughput applications [13,14,15]. Already 20 years ago it was shown that spheroidal cultures of breast cancer cells behave completely differently through the additional dimension [16]. Subsequently, spheroid models of various organ systems such as the intestine [17], brain [18] or liver [19] were successfully established to bridge the large gap between monolayer cell culture and living tissue [13]. Today, it is generally accepted that spheroid cultures much better mimic an in vivo-like microenvironment by ensuring physiological cell–cell and cell–ECM interactions. While Kale et al. paved the way for further research on osteoblast spheroids by showing the importance of a 3D cellular development for ex vivo bone formation [20], limitations included the dependence on cytokine supplementation and a huge size variability between spheroids. Limited other research also provided evidence that the cellular response of osteoblast(-like) cells cultured in 3D substantially differs from that in 2D [21,22]. A major limitation of currently scarcely reported approaches is that mineralization was either not reported [21,22,23,24] or has not (reliably) been quantified [25,26]. Yet, no generally accepted spheroid model for osteoblast(-like) cells has been established so far.

Interestingly, commercially available MC3T3-E1 osteoprogenitor cells are well-established and undergo a synchronized osteoblastic differentiation in vitro [27] and even enhance bone healing in vivo [28,29]. MC3T3-E1 cells express high amounts of alkaline phosphatase, produce minerals, and are capable of differentiating into osteocytes. This makes them appropriate for studying terminal osteogenesis [30] and an excellent model of primary osteoblasts when treated with osteogenic differentiation media [31]. During intramembranous osteogenesis, pre-osteoblasts are formed from progenitor cells which, by a so-called condensation, progress towards differentiating pro-osteoblastic cell aggregates [32]. Osteogenic differentiation in vitro is thus a tightly regulated process, characterized by a sequential gene expression of well-recognized molecular markers. *Runx2* expression is usually high during the pre-osteoblastic stage [33], while mature osteoblasts are characterized by a relatively lower expression level of *Col1a1* and a higher expression of, e.g., alkaline phosphatase (*Alp*), secreted phosphoprotein 1 (*Spp1*; syn.: osteopontin), and bone gamma-carboxyglutamate protein (*Bglap*, osteocalcin). Type I collagen (*Col1*) is considered an early osteoblast differentiation marker and increased expression is observed during the transformation of osteoprogenitor to pre-osteoblasts [34]. Terminally differentiated osteocytes suppress *Alp*, *Col1a1*, and *Bglap* expression, only maintaining *Spp1* expression levels [35]. Osteocalcin, on the other hand, is a small conserved non-collagenous extracellular matrix protein expressed during late osteoblastic differentiation and abundantly expressed in bone [36]. Its function is associated with the mineralization and matrix synthesis and is thus considered a late differentiation marker [37].

However, for a routine pharmacological in vitro assay to screen drugs for their potency in altering osteogenesis or biomineralization, achieving quantifiable calcium deposition is utmost important. Notably, Alizarin red staining and von Kossa staining are two widely, and largely alternatively, used but distinctly different and inexpensive techniques to semi-quantitatively examine mineralization in vitro. Their appropriateness to demonstrate bone-like biomineralization, however, has lately been questioned [38]. On the other hand, micro-computed tomography (micro-CT) has become a routine technique to noninvasively quantify bone mineralization dynamics [39].

This study thus aimed to develop a versatile biomimetic, and high-throughput compatible, robust mammalian progenitor cell-based spheroid in vitro culture system to study and reliably quantify physiological biomineralization processes using well-established murine MC3T3-E1 osteoprogenitor cells in a modified forced-floating methodology.

## 2. Materials and Methods

**Cell culture** Murine MC3T3-E1 pre-osteoblasts were grown at 37 °C and 5% CO_2_ in alpha-minimum essential medium (α-MEM; Sigma, St. Louis, MO, USA), supplemented with 10% fetal calf serum (FCS; ThermoFisher Scientific, Waltham, MA, USA), 1% (*w*/*v*) L-glutamine (Sigma, St. Louis, MO, USA), and 1% (*w*/*v*) penicillin/streptomycin (P/S; Sigma, St. Louis, MO, USA), referred to as α-MEM+, which was changed every second day. For 2D cultures, after reaching about 80% confluence, cells were trypsinized, resuspended, and seeded into 12-well plates at 71,450/cm^2^ (i.e., 250,000/well) and allowed to adhere overnight prior to medium change. For 3D cultures, trypsinized cells were resuspended in α-MEM+, with 1× NEAA (Sigma, St. Louis, MO, USA) instead of L-glutamine, and 250,000 cells/250 µL pipetted into non-adhesive v-shaped 96-well plates (Nunc^TM^MicroWell^TM^, ThermoFisher Scientific, Waltham, MA, USA), centrifuged at 300× *g* for 8 min, and 100 µL of fresh α-MEM+ being added after 24 h. With medium changes every other day, cells were cultured for up to 21 days or 28 days, respectively, in either α-MEM+ control medium (CM) or in differentiation medium (DM), additionally containing 10 mM b-glycerophosphate (BGP; Sigma, St. Louis, MO, USA) and 50 µg/mL ascorbic-acid-2-phosphate (Sigma, St. Louis, MO, USA). In certain experiments, differentiation medium (DM) without or with lithium chloride (5 mM; Sigma-Aldrich, St. Louis, MO, USA) was used.

**DNA assays** Total DNA content was determined using a Quant-iT™ PicoGreen™ dsDNA Assay Kit (ThermoFisher Scientific, Waltham, MA, USA) according to the manufacturer’s protocol. Briefly, after cell lysis in 1× passive lysis buffer (Promega, Fitchburg, WI, USA), a 50 µL sample was incubated with an equal volume of reagent solution for 5 min at RT in the dark. Then, fluorescence was measured on a microplate reader (Infinite M200, TECAN, Salzburg, Austria) at an excitation wavelength of 480 nm and an emission wavelength of 520 nm. The total amount of DNA was calculated from the fluorescence values of a standard curve of known lambda DNA concentrations provided by the kit and expressed as ng per spheroid.

**Nucleic acid isolation and gene expression analyses** Briefly, three spheroids were pooled per replicate and homogenized in a Precellys^®^ Tissue Homogenizer (Bertin GMBH, Frankfurt am Main, Germany) prior to peqGOLD Trifast^TM^ (Peqlab, Erlangen, Germany) isolation of total RNA according to the manufacturer’s instructions. RNA concentration and purity (A_260_/A_280_ & A_260_/A_230_) were determined spectrophotometrically on a NanoDrop1000 (Peqlab, Erlangen, Germany), as reported earlier [40]. Upon digestion of genomic DNA with Ambion^TM^ DNase (ThermoFisher Scientific, Waltham, MA, USA), cDNA synthesis was performed using mixed priming (oligo-dT/random hexamer) and Maxima Reverse Transcriptase (RT; ThermoFisher Scientific, Waltham, MA, USA). RT-qPCR was carried out as 15 μL reactions with 2 μL cDNA (equates to 0.8 ng cDNA), 7.5 μL PowerSYBR^TM^ Green PCR Master Mix (Thermo Fisher Scientific, Waltham, MA, USA), 4 µL DEPC water, and 0.75 μL of each primer (500 pM). PCR was performed in a StepOnePlus cycler (Thermo Fisher Scientific, Waltham, MA, USA) with an initial 10-min denaturation at 95 °C, 40 amplification cycles for 15 s at 95 °C, annealing for 30 s (see Table 1 for individual annealing temperatures), and 30 s of elongation at 72 °C with fluorescence measurements at the end of each cycle. Final denaturation (95 °C for 15 s) was followed by a melting curve from 60 °C to 95 °C. Samples were measured in technical duplicates with nuclease-free water as a non-template control. Amplification efficiencies were determined using LinRegPCR 2016 software (Academic Medical Centre, Amsterdam, The Netherlands) and relative gene expression was calculated after normalization to previously defined reference genes (mHprt, mSdha) according to a geNorm study, which was conducted prior the gene expression study. Qbase+ 3.3 software (Biogazelle, Gent, Belgium) was used to calculate relative changes in target gene expression according to an efficiency-corrected ΔΔCq method [41,42].

**Extracellular calcium assay** Bound calcium was dissolved from the extracellular matrix (ECM) by incubating PFA-fixed spheroids overnight in 100 µL of 0.5 M acetic acid under gentle shaking. Calcium concentration in the supernatant was determined using a Calcium Assay kit (abcam, Cambridge, UK) according to the manufacturer’s protocol. Briefly, 50 µL of each sample was incubated with 90 µL of a chromogenic solution and 60 µL of the calcium detection buffer for 10 min at room temperature (RT) in the dark. Absorbance was measured at 575 nm using a spectrophotometer (Infinite M200, TECAN, Salzburg, Austria).

**Determination of ALP activity** Cells were briefly washed once with 1× PBS before lysis in 210 µL per well of a 12-well plate, or 150 µL per spheroid, of 1× Passive Lysis Buffer (PLB), respectively. Specimens were incubated for 30 min at RT under vigorous shaking and frozen at −80 °C. This was followed by two additional thawing/freezing steps. Lysates (2D) were diluted 1:10 with 1× PLB, while spheroid lysates were used undiluted. Finally, 50 µL of the working lysate was mixed with 50 µL of the *p*-nitrophenol solution (1 mg *p*-nitrophenyl phosphate per mL of 1 M diethanolamine, pH 9.8; Merck KGaA, Darmstadt, Germany) in a 96-well microtiter plate. After incubation at 37 °C for 10 min, the reaction was stopped with 100 µL of a 0.1 M NaOH and absorbance was measured at 405 nm in technical triplicates in a spectrophotometer (Infinite M200, TECAN, Salzburg, Austria) to calculate DNA-normalized ALP activity, expressed as units/ng DNA.

### 2.1. Histochemistry and Immunohistochemistry

#### 2.1.1. 2D Cultures

**Alizarin Red staining** to determine mineralization was performed after 21 days of culturing according to the method described by Gregory et al. [43]. Therefore, cells were washed with 1× PBS before fixation with 1 mL of 4% formalin solution for 1 h at RT. Subsequently, samples were washed twice with ultrapure water and then stained with 2% Alizarin Red solution for 45 min at RT. Excess dye was removed by rinsing four times with ultrapure water before taking photographs. For quantification of the staining, the dye bound to calcium was extracted with acetic acid. Therefore, 400 µL of 10% acetic acid was added to each well, followed by incubation for 30 min under gentle shaking. Cells were resuspended using a cell scraper and the contents of each well were transferred into a 1.5 mL Eppendorf tube. The lids of the tubes were sealed with parafilm to prevent evaporation during the subsequent heat treatment at 85 °C for 10 min under shaking. Samples were then allowed to cool on ice for 5 min prior to centrifugation at 20,000× *g* for 15 min. 200 µL of the supernatant was transferred to a new Eppendorf tube and mixed with 75 µL of a 10% ammonium hydroxide solution. 50 µL was added to a 96-well microtiter plate and absorbance was measured in technical triplicates at 405 nm.

**Sirius Red staining** of monolayers was performed after 21 days of culture using a previously described modification of the method of Tullberg-Reinert et al. [44,45]. First, cells were washed with 1× PBS and then fixed with Bouin’s solution for 1 h at RT. After washing three times with ultrapure water, the samples were stained with 0.1% Sirius red dye solution for 1 h at RT. Then, photographs of the wells and microscopic images of the cells were taken. Subsequently, the samples were washed first with 0.5 M acetic acid and then with ultrapure water. Extraction of the bound dye was performed with 300 µL of 0.1 M NaOH for 30 min under gentle shaking. Fifty µL of the sample was pipetted into a 96-well microtiter plate and absorbance was measured at 590 nm in technical triplicates.

#### 2.1.2. 3D Cultures

**Hematoxylin–Eosin staining** spheroids were routinely embedded in paraffin [46], with modification described earlier by us for 3D cultures of expended chondrocytes [47]. After deparaffinization, overview staining with hematoxylin-eosin (HE) of decalcified spheroid sections (3 µm) was performed. For this purpose, sections were placed in hemalaun solution (Merck KGaA, Darmstadt, Germany) for 8 min, rinsed with tap water for 5 min, and stained with eosin (Merck KGaA, Darmstadt, Germany) for 2 min.

**Alizarin Red staining** undecalcified spheroid sections were stained for 5 min with a 2% Alizarin Red S (Sigma, St. Louis, MO, USA) solution (pH 4.2). Non-decalcified spheroid sections were stained according to von Kossa by incubating them for 1 h in a 5% silver nitrate solution under a UV lamp. After three washes with ultrapure H_2_O, the sections were placed in a 5% sodium thiosulphate solution for 5 min. The sections were washed three times with ultrapure water before the nuclei were counterstained with a nuclear red solution (Merck KGaA, Darmstadt, Germany) for 5 min.

**Sirius Red staining** collagen was stained histochemically by incubating the decalcified spheroid sections in 0.1% Sirius Red solution for 60 min and then rinsing them in two changes of 0.5% acetic acid water. The Sirius Red solution consisted of 0.1% Direct Red 80 (Sigma, St. Louis, MO, USA) dissolved in a saturated aqueous solution of picric acid.

**TUNEL/Ki67 co-staining** after deparaffinization of the decalcified spheroid sections, antigens were unmasked in 0.01 M citrate buffer, pH 6.0 at 600 W in the microwave for 15 min. Sections were blocked with PBS/10% horse serum/1% BSA for 60 min at RT before incubation with the diluted primary anti-Ki67 antibody overnight at 4 °C (Invitrogen, MA5-14520, Waltham, MA, USA, 1:200 in PBS/1.5% BSA). Sections were washed 3 times in PBS and the diluted secondary donkey anti-rabbit antibody (Alexa Fluor 546, Life Technology A 10,040, Carlsbad, CA, USA, 1:2000 in PBS/1.5% BSA) was applied for 1 h at RT in the dark. Slides were washed 3 times with 1× PBS, and apoptotic cells were stained using the In Situ Cell Death Detection Kit (Roche Diagnostics GmbH, Mannheim, Germany) according to the manufacturer’s protocol. Briefly, each section was stained with 50 µL of the TUNEL reaction mixture and incubated at 37 °C for 60 min. Slides were washed twice with 1× PBS and nuclei were counterstained with bisbenzimide (conc.: 1 ng/mL; Sigma, St. Louis, MO, USA) for 10 min.

**Immunohistochemistry** after deparaffinization of the decalcified spheroid sections, the slides were incubated in a methanol solution containing 3% hydrogen peroxide at RT for 15 min to block endogenous peroxidase. Slides were washed twice with 0.05 mM Tris-buffered saline (TBS), pH 7.6 before antigen retrieval in 0.01 M citrate buffer, pH 6.0 at 65 °C for 30 min. After blocking in TBS/1.5 % BSA/2 % milk powder, the sections were incubated overnight at 4 °C with primary antibodies. Antibodies were diluted in TBS/1.5% BSA as follows: anti-collagen type I 1:200 (Invitrogen, Waltham, MA, USA), anti-osteocalcin 1:150 (Bioss, Woburn, MA, USA), anti-osteopontin 1:100 (Abcam, Waltham, MA, USA), anti-Runx2 1:100 (Santa Cruz, Dellas, TX, USA). Samples were washed twice with TBS before incubation with the secondary antibody, conjugated to the HRP-labelled polymer (Dako, Carpinteria, CA, USA) for 30 min at RT. Visualization was performed with an AEC kit (Invitrogen, Frederick, MO, USA) for 5 min and nuclei were counterstained by incubation in hemalaun.

**Micro-CT Examination** the PFA-fixed spheroids were positioned in the sample holder of the micro-CT (Skyscan 1176, Bruker micro-CT, Kontich, Belgium) and scanned with the following settings: 60 kV, 0.25 mm aluminum filter, 166 µA source current, exposure time 1166 msec, 5 µm isotopic resolution, 8 projection images per 0.025° rotation step, rotation range 360°. Volumetric data were reconstructed using NRecon/InstaRecon CBR Server CPremium software (Skyscan, Kontich, Belgium/InstaRecon, Champaign, IL, USA). Imalytics Preclinical software (Gremse-IT, Aachen, Germany) was used for image analysis, segmentation of micro-CT data, and quantification of mineralization [48]. Areas of the spheroid with a voxel intensity of 45,000 (arb. Unit) were defined as highly mineralized, whereas areas with a voxel intensity between 35,000 and 44,999 (arb. Unit) were characterized as moderately mineralized.

**FTIR Analysis** the PFA-fixed spheroids and a 2 × 2 × 2 mm specimen of a murine femur were washed extensively with PBS and allowed to air dry. The samples were measured with the Alpha IR (Bruker, Billerica, MA, USA) and analyzed with the spectroscopy software OPUS (Bruker, Billerica, MA, USA).

**Scanning Electron Microscopy** for scanning electron microscopy, the PFA-fixed samples were washed in PBS for 15 min and were dehydrated in an ascending ethanol series (30%, 50%, 70%, 90% and 100%) for 10 min each and three times during the last step. The samples were critical-point-dried in liquid CO_2_ (Polaron, GaLa Instrumente, Bad Schwalbach, Germany) and sputter-coated with a 10 nm thick gold/palladium layer (Sputter Coater EM SCD500; Leica, Wetzlar, Germany). The samples were observed under an environmental scanning electron microscope (ESEM XL 30 FEG, FEI, Eindhoven, The Netherlands) equipped with an EDX detector system (EDAX Genisis System) with an accelerating voltage of 10 kV.

**Statistics** variance homogeneity was tested using the Brown-Forsythe test (*p* > 0.05). The Shapiro–Wilk test was applied to examine the normal distribution of the residuals (*p* > 0.05). Where necessary, a BoxCox-Y transformation or a Johnson transformation was performed to achieve homoscedasticity. For analysis of parametric data, a Student’s *t*-test was performed to compare 2 groups, and one-way or two-way ANOVA followed by Tukey’s post hoc test was conducted for multiple comparisons. Non-parametric data were analyzed using the Kruskal–Wallis test followed by the Dunn’s post hoc test. All data are presented in terms of geometric mean ± 95% CI and P value less than 0.05 was considered significant. Statistical analyses were performed using GraphPad Prism 9 (GraphPad, La Jolla, CA, USA) and JMP 10 (Böblingen, Germany).

## 3. Results

### 3.1. Biomineralization in Spheroids

**Figure 1:**
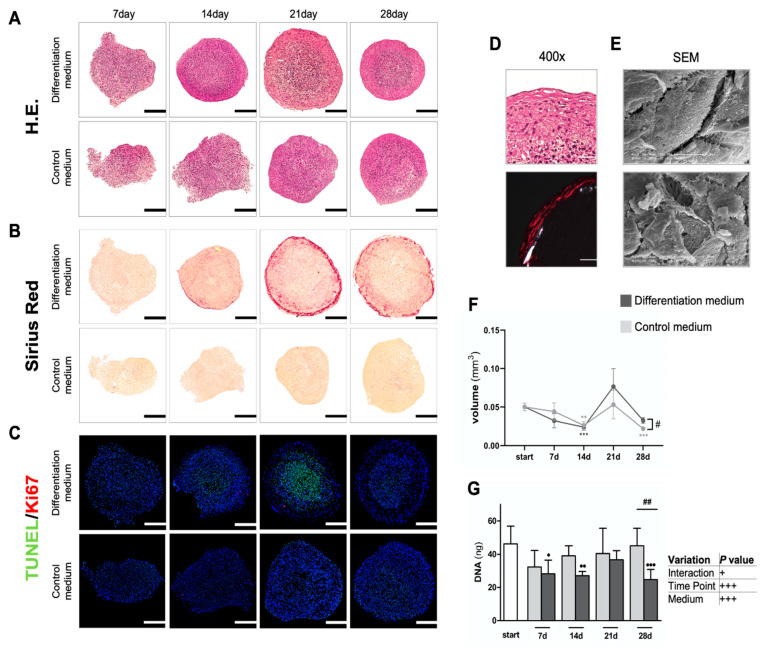
**General microscopic appearance of spheroids, next to collagen content and cell death.** (**A**) Hematoxylin–Eosin (H.E.) overview of staining, showing nuclei in violet blue and cytoplasm and ECM in shades of pink. (**B**) Sirius Red staining of collagens. (**C**) TUNEL/Ki67 co-staining to detect apoptotic cell death and proliferation. Representative stainings are shown (*n* = 3), scale bars represent 200 µm. (**D**) 400× *g* magnifications of the rim region of HE-stained differentiating spheroids at day 28 with polarized light micrograph of Sirius Red staining underneath. Scale bars are 50 µm. (**E**) Representative SEM images of the outer spheroid layer, cultured for 28 days in differentiation medium. (**F**) Spheroid volume determination by micro-CT. Significant differences were calculated by 2-way ANOVA (*n* = 6) followed by Tukey’s post hoc test of BCY-transformed data. (**G**) Total DNA analyses of the spheroids using BCY-transformed data with two-way ANOVA (*n* = 6), followed by Tukey’s post hoc test, next to tabular overview. ^#^ *p* < 0.05, ^##^ *p* < 0.01 as indicated; ^•^ *p* < 0.05, ^••^ *p* < 0.01, ^•••^ *p* < 0.001 vs. starting day. Data represent geometric mean ± 95% CI.

Spheroids from MC3T3-E1 osteogenic progenitor cells were reproducibly obtained by a modified forced floating method. In this novel physiological in vitro biomineralization model, cells formed spheroids within two days upon seeding. As evident from representative HE-stained overview sections (Figure 1A), spheroids were initially less uniformly shaped. However, spheroids showed only minor variability of their respective total volumes at the starting point (i.e., day 2 after seeding) of their cultivation (Figure 1F), as objectively determined by micro-CT analyses. By day 14, spheroids in differentiation medium reached a stable spherical morphology, while the controls did not reach a stable spherical shape until day 21 (Figure 1A). Interestingly, shrinkage of spheroids in both culturing conditions during the first 14 days was significant when compared to the starting point (*p* < 0.001 vs. *p* < 0.01 for differentiation and control medium, respectively) (Figure 1F). Cellular condensation with the formation of a cell-enriched core regions began in spheroids under differentiation conditions from day 14 onwards. The formation of a fibrous capsule, with elongated fibroblast-like cells, was evident at late culture stages under osteoinductive conditions (Figure 1A,D). Deposition of collagen fibers in the capsule of differentiating spheroids was confirmed by Sirius Red (Figure 1B). Notably, 400× *g* magnifications of the rim region of HE-stained differentiating spheroids at day 28, and corresponding representative polarized light micrographs, underscore this finding (Figure 1D). Electron micrographs from the same area revealed an elongated cell shape and a collagen fibril network in the spheroid capsule (Figure 1E). Eventual cell death due to apoptosis and any changes in cell numbers due to proliferation were assessed by TUNEL/Ki67 co-staining (Figure 1C) and by determination of DNA content per spheroid (Figure 1G), respectively. Proliferation was not significantly affected at any time point in any condition, despite the presence of some Ki67-positive cells, on day 14 and day 21, in differentiating spheroids in a small area between the core region and the fibrous outer rim of the spheroids (Figure 1C). Apoptosis, or any changes in DNA content, was insignificant under control conditions (Figure 1G). In contrast, starting from day 14, and peaking at day 21, TUNEL staining detected numerous apoptotic cells in the condensed core region of differentiating spheroids (Figure 1C). Total cell numbers per spheroid also differed between both conditions by day 28 (Figure 1G), which indirectly hints towards a relatively increased rate of apoptosis in differentiating spheroids. Despite their lower DNA content, differentiating spheroids revealed a significant volume increase by day 28 (*p* < 0.05) (Figure 1F,G), which may suggest enhanced ECM synthesis under osteoinductive conditions.

#### 3.1.1. Osteogenic Differentiation of Spheroids

**Figure 2:**
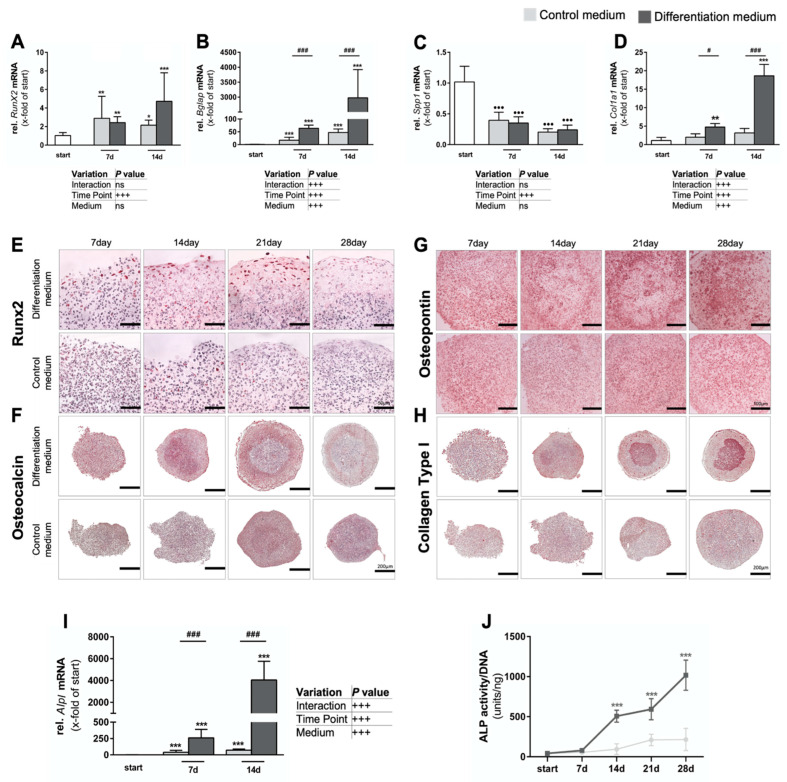
**Expression of osteogenic markers**. (**A**–**D**) Relative mRNA expression of selected differentiation markers during the first 14 days of culture, with tabular summary of analyses underneath. Shown are *Runx2* (**A**), *Bglap* (osteocalcin) (**B**), *Spp1* (osteopontin) (**C**), and *Col1a1* (**D**). Significant differences were calculated by 2-way ANOVA (*n* = 5), followed by Tukey post hoc test. (**E**–**H**) Corresponding immunohistology. IHC staining against Runx2 (**E**), osteocalcin (**F**), osteopontin (**G**) and type I collagen (**H**). Representative staining from at least three spheroids (*n* = 3) per condition; scale bars as indicated. (**I**) Changes in *Alpl* mRNA abundance until day 14 of culture and (**J**) respective enzymatic activity over 28 days. Significant differences were calculated by 2-way ANOVA (*n* = 5) of BCY-transformed data followed by Tukey’s post hoc test (**I**) and Kruskal–Wallis (*n* = 6) followed by Dunn’s post hoc test (**J**). ^#^ *p* < 0.05 ^###^ *p* < 0.001 as indicated; * *p* < 0.05, ** *p* < 0.01, *** *p* < 0.001, ^•••^ *p* < 0.001 vs. starting day. Data represent geometric mean ± 95% CI.

Next, we determined mRNA expression profiles of selected early and late osteoblast differentiation markers during the first 14 days of spheroid culture. Gene expression level of early osteogenic differentiation marker *Runx2* changed culture time-dependently (Figure 2A) and specifically increased from day 7 to day 14 under differentiating conditions as opposed to the controls (*p* < 0.05 and *p* < 0.001, respectively). Corresponding immunohistochemical (IHC) staining failed to detect a significant difference in RUNX2-positive cells between both conditions at day 7 (Figure 2E), which might have been due a lag phase between transcription and translation and an overall limited number of RUNX2-positive cells at this time point. From day 14 on, and peaking at day 21, IHC revealed plenty of RUNX2-positive cells in an area between the condensed core and the outer fibrous capsule of differentiating spheroids, whereas RUNX2 protein expression was virtually absent in controls.

Osteocalcin (*Bglap*) gene expression levels significantly increased over the culture period (*p* < 0.001), and specifically under osteogenic conditions, reaching a 63-fold increased mRNA abundance above control conditions by day 14 (*p* < 0.001) (Figure 2B). While, as compared to the starting point, *Bglap* expression also increased on days 7 and 14 (*p* < 0.001) in control cultures, IHC identified a strong protein expression on day 14 specifically in differentiating spheroids (Figure 2F), which is in line with the expectation. Interestingly, by that time, osteocalcin expression filled the entire condensed core region. However, this expression pattern was not confirmed at days 21 and 28, respectively. Only in the small rim region between the core and the outer fibrous capsule some osteocalcin-positive staining remained. To our knowledge such a strikingly spaciotemporal expression pattern has never been reported in 3D cultures before. Osteocalcin protein expression was undetectable in controls at all time points.

From day 7 on, osteopontin (*Spp1*) gene expression decreased over time (*p* < 0.001) relative to the starting point (Figure 2C), independent of the medium type. At later time points, IHC initially showed a ring-shaped osteopontin expression pattern at the edge of the condensed core area of differentiating spheroids (Figure 2G) until the entire core was positively stained on day 28.

Differentiation conditions also upregulated *Col1a1* expression (*p* < 0.001) as compared to controls (Figure 2D) and also over the course of the culture period (*p* < 0.001). Of note, *Col1a1* expression was already significantly upregulated (*p* < 0.05) at day 7, reaching an about 5.9-fold higher expression level in differentiating conditions by day 14 (*p* < 0.001). IHC confirmed type I collagen deposition from day 14 on, especially in the center of differentiating spheroids, from where it radially expanded outwards until the entire core region was positively by day 28 (Figure 2H). In contrast, the fibrous capsule of these spheroids remained negative for type I collagen and control spheroids expressed only minor amounts of type I collagen over the entire culture period.

Alkaline phosphatase, a well-established osteogenic differentiation marker of mesenchymal stem cells [30], was also analyzed to determine gene expression (Figure 2I) and enzyme activity (Figure 2J) level. In both instances, culture period and osteoinductive conditions significantly regulated their mRNA levels (*p* < 0.001) (Figure 2I). On both day 7 and day 14, *Alpl* gene expression was significantly increased (*p* < 0.001) under differentiating conditions as compared to control spheroids, with a more than 55-fold peak increase on day 14. However, *Alpl* expression also increased between starting point and day 7 and day 14, respectively, in controls. DNA-normalized enzyme activities also significantly increased from day 14 on until the end of culture (*p* < 0.001), in differentiating spheroids as compared to their starting point (Figure 2J). In contrast, ALP activity did not change in the controls. 

#### 3.1.2. Intense Biomineralization in Osteogenic Spheroid Cultures

**Figure 3:**
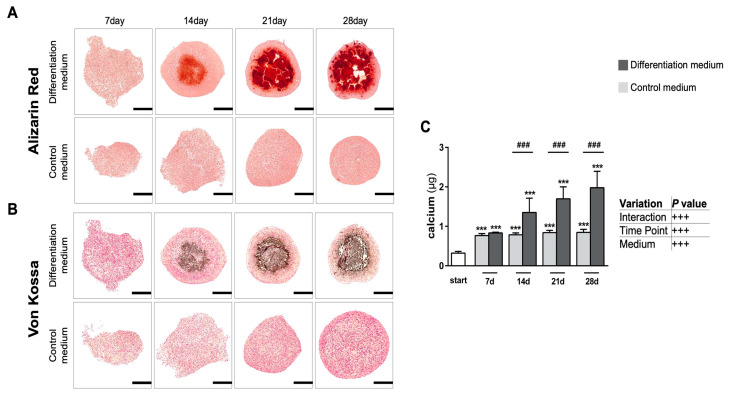
**Qualitative and quantitative evaluation of calcium deposition within spheroids.** (**A**) Alizarin Red staining over a culture period of up to 28 days. Note that Alizarin sulfonic acid stains calcium deposits deep brick orange-red. (**B**) Corresponding von Kossa staining, showing calcifications in greyish-black. Note that calcium deposition is not observed in non-differentiating controls. Representative images from sectioning three randomly selected spheroids (*n* = 3) per condition. Scale bars are 200 µm. (**C**) Spectrophotometric quantification of the extracted precipitated calcium at 405 nm. Significant differences were evaluated using a two-way ANOVA of the Johnson transformed data (*n* = 6, mineralizing groups; *n* = 5, control groups), followed by Tukey’s post hoc test. ^###^ *p* < 0.001 as indicated; *** *p* < 0.001 vs. starting day. Data represent geometric mean ± 95% CI.

Alizarin red S (ARS) precipitates Ca^2+^ ions to form brick-red deposits through its sulfonate and hydroxyl groups (Figure 3A); von Kossa (Figure 3B) is a widely used histological staining method which detects the presence of abnormal calcium deposits, which it stains greyish-black. We thus subsequently collected histochemical evidence of mineralization in the condensed spheroid cores that were cultured under differentiating conditions. Generally, mineralization always began centrally in the core region of the spheroids after about 14 days of culture and extended radially outwards until almost the entire spheroid cross-section was strongly positively stained by day 28 in mineralizing conditions. Usually, only the fibrous outer capsule and a thin area underneath it remained unmineralized (Figure 3A,B). In contrast, no mineralization was detectable at any time point in spheroids grown in the non-mineralizing control medium (Figure 3A,B). Beyond this semi-quantitative approach, we further quantified the extent of calcium deposition by acid-based extraction, followed by spectrophotometric determination of the chromophoric calcium–cresolphthalein complexes to confirm earlier histochemical observations (Figure 3C). From day 14, osteogenic culture conditions led to significantly higher calcium accumulations than in control spheroids (*p* < 0.001). This accumulation progressively continued over the whole culture period in differentiating spheroids (*p* < 0.001) (Figure 3C).

**Figure 4:**
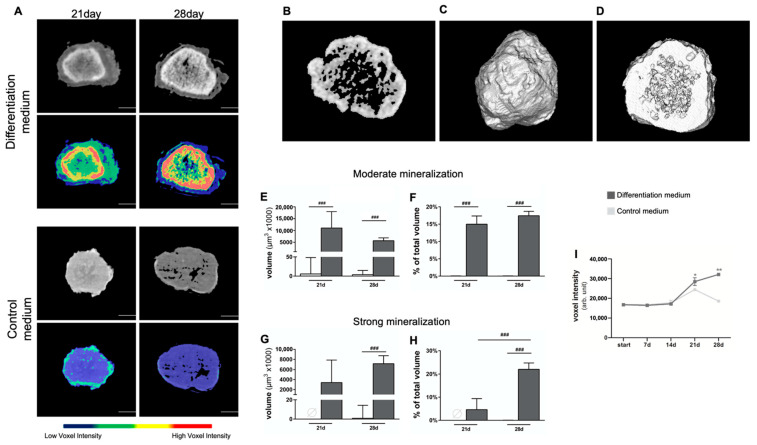
**Volumetric quantification of biomineralization in spheroids by micro-CT.** (**A**) On top, original micro-CT image; below, pseudo-colored image after segmentation by voxel intensity. Representative 2D (**B**) and 3D (**C**) reconstruction with corresponding cross-section (**D**) showing the mineralized core of a spheroid cultured for 28 d under osteogenic conditions. Note the central trabecular-like mineralization pattern surrounded by a ring-shaped mineralization. (**E**–**H**) Micro-CT-based volumetric quantification of biomineralization, with arbitrarily chosen pixel intensity cut-off values, discriminating moderate (**E**,**F**) and strong mineralization (**G**,**H**) by objective differences in the central calcium deposit volume. Significant differences were evaluated using a one-way ANOVA (*n* = 6, mineralizing groups; *n* = 5 control groups), followed by Tukey’s post hoc test. (**I**) Changes in mean voxel intensities over time. Statistical differences were calculated by Kruskal–Wallis method followed by Dunn’s post hoc test. ^###^ *p* < 0.001 as indicated; * *p* < 0.05, ** *p* < 0.001 vs. starting day. Data represent geometric mean ± 95% CI.

Additionally, changes in radiopacity due to mineralized tissue formation in spheroids between conditions and over the culture period was volumetrically and quantifiable examined by micro-CT analyses. Based on actual voxel intensities, gray-level thresholding generated pseudo-colored gradients, allowed cut-off value-based binning into moderately (yellow areas, Figure 4A) and strongly mineralized regions within a spheroid (red areas, Figure 4A). Consistent with our previous findings, greater volumes of both moderately mineralized (*p* < 0.001, on days 21 and 28) and strongly mineralized (*p* < 0.001, on day 28; on day 21 control spheroids = 0, excluding statistical comparison) regions were detected in spheroids in differentiating conditions (Figure 4E,G). The percentages of highly mineralized regions (Figure 4H) of the total spheroid volume increased significantly over time, from day 21 to day 28 (*p* < 0.001), respectively. Moderate or strong mineralization was mainly found in a ring-shaped region at the transition zone to non-mineralized regions in the spheroids from day 21 on, while the core regions showed a much lower radiopacity (Figure 4A,B,D). On day 28, trabeculae-like patterns of more intense radiopaque mineralization were observed in more central areas (Figure 4B,D). Thus, while histochemistry suggests a more or less homogenous, uniform mineralization of the entire spheroid core region (Figure 3A,B), micro-CT examination allowed the discrimination of different levels of mineralization intensities as well as detection of refined distribution patterns within the same spheroid (Figure 4A,B,D). Consistent with our previous data, determining mean voxel intensities further revealed that differentiating spheroids accumulated more calcium deposits and thus obtained a significantly higher radiopacity at day 21 (*p* < 0.05) and 28 (*p* < 0.01) as compared to the starting point of the culture (Figure 4F).

#### 3.1.3. Formation of Native Hydroxyapatite-like Biomineralization in Spheroid Cultures

**Figure 5:**
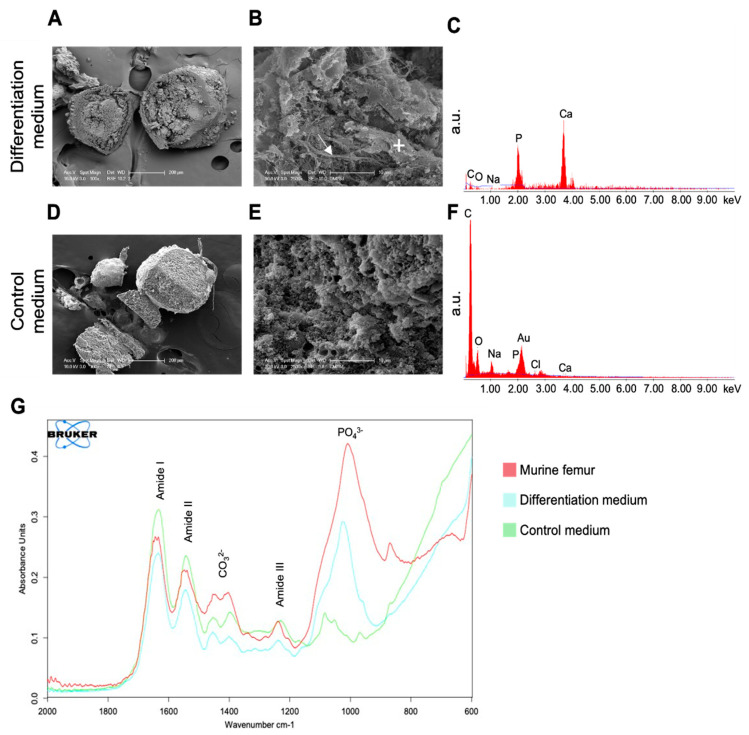
**Native bone-like biomineralization in 3D cultures.** (**A**–**C**) Representative SEM/EDX analysis of a spheroid cultured for 28 days in differentiation medium, with cross-section (**A**), magnification (**B**), and EDX spectrum (**C**). (**D**–**F**) Representative corresponding SEM and EDX analyses of a spheroid cultured under non-mineralizing conditions. (**G**) Representative FTIR absorption spectra of a murine cortical bone specimen (i.e., femur, red), and spheroids cultured for 28 days under either mineralizing (blue) or non-mineralizing culture conditions (green). a.u., arbitrary units; arrow marks a collagen fiber; “+” marks a cell.

After thorough determination of quantitative differences in the mineralization patterns in osteogenic spheroid cultures by aforementioned techniques, we next set out to characterize quality of the mineralization by energy-dispersive X-ray spectroscopy (EDX) and Fourier transform infrared spectroscopy (FTIR). First, critical-point dried spheroids were examined by scanning electron microscopy (SEM) (Figure 5A–F). Of note, after 28 days of culture, the mineralized core region and the fibrous capsule are identifiable in a cross-sectioned spheroid (100× overview images, Figure 5A vs. D). In higher magnifications (2500× *g*, Figure 5B,E) of mineralized cores upon cultivation in differentiation medium, a collagenous network formation is visible. Note the collagen fibers in which MC3T3-E1 cells are embedded (Figure 5B, see legend for details). In contrast, control spheroids from non-mineralizing conditions reveal a rather homogeneous cross section in the overview image (Figure 5D) without apparent collagen fibers (Figure 5E).

Next, we used EDX for an elemental analysis of the chemical composition of the mineral deposits (Figure 5C,F). EDX spectra of the mineralized core regions of a spheroid cultured for 28 days in differentiation medium clearly showed an enrichment of calcium (Ca) and phosphate (P) (Figure 5C). In contrast, such an accumulation was absent in control spheroids (Figure 5F).

As FTIR offers qualitative and quantitative analysis through infrared absorption spectra, providing a distinctive molecular fingerprint of the sample, we performed FTIR analyses on spheroids grown for 28 days in control medium (green line) or differentiation medium (blue line) (Figure 5G). As reference material, a piece of cortical bone from a mouse femur (red line) was used. Representative spectra, between 600–2000 cm^−1^, are shown in Figure 5G. All samples show spectral peaks at 1650 cm^−1^, 1550 cm^−1^, and 1240 cm^−1^, matching the infrared spectra of amides I, II, and III, providing the typical absorption fingerprint of an organic matrix. In addition, FTIR analyses further revealed the presence of characteristic phosphate groups (spectral range between 900–1200 cm^−1^) exclusively in the differentiated spheroids and the mouse femur reference. Thus, from the almost congruent IR fingerprints between the latter samples, it appears that differentiated spheroids synthesize an almost native bone-like matrix.

### 3.2. Comparing Effects of Pharmacological cWnt Agonist Lithium in 2D vs. 3D

To test our hypothesis that pharmacological compounds differentially affect 2D monolayer and biomimetic 3D spheroid cultures, we evaluated the effect of lithium chloride on the osteogenic differentiation of MC3T3-E1 cells. The best characterized pharmacological effect of lithium is the inhibition of glycogen synthase kinase-3β and consequently the activation of canonical Wnt (cWnt) signaling [11].

**Figure 6:**
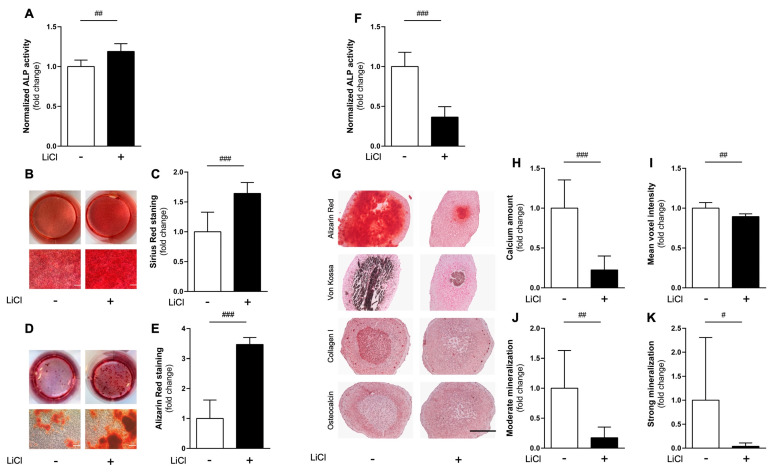
**Different responses to cWnt agonist in monolayer and spheroid cultures.** (**A**–**E**) Effect of LiCl on MC3T3-E1 cells cultured in 2D: determination of alkaline phosphatase (ALP) activity after 14 days of culture without (-) or with (+) 5 mM LiCl (**A**). Representative Sirius Red stainings after 21 days with macroscopic photographs of wells (top) and respective micrographs (bottom) (**B**) and corresponding spectrophotometric quantification of total collagen content upon dye extraction (**C**). Corresponding Alizarin Red staining (**D**) with quantification of calcium deposition (**E**). Scale bars show 100 µm. (**F**–**K**) Comparison of lithium-mediated effects in 3D-cultured MC3T3-E1 cells. ALP activity after 14 days (**F**), representative immuno-/histochemically stained sections (i.e., Alizarin Red, Von Kossa, type I collagen, osteocalcin, from top to bottom) after 21 days. Quantified extracellular calcium content in spheroids after 21 days (**H**) and corresponding micro-CT analyses: mean voxel intensity (**I**), volumetric quantification of moderate (**J**) and strong mineralization levels (**K**). For details see legend to Figure 3. Scale bars are 200 µm. Significant differences were evaluated using Student’s *t*-test (*n* = 6). ^#^ *p* < 0.05, ^##^ *p* < 0.01, ^###^ *p* < 0.001 as indicated. Data represent geometric mean ± 95% CI.

In both 2D and 3D cultures, cells were maintained for up to 21 days either with or without the addition of 5 mM lithium chloride. This concentration was chosen based on results from earlier exploratory studies (data not shown) and in agreement with values from the literature. At this concentration, lithium was earlier shown to promote differentiation and mineralization in 2D-cultured MC3T3-E1 cells [49], which was confirmed by our 2D results (Figure 6A–E): lithium significantly enhanced alkaline phosphatase (Alp) activity (*p* < 0.01) after 14 days as compared to cells cultured without lithium (Figure 6A). MC3T3-E1 cells in monolayers further showed a higher collagen deposition after 21 days of culture as evident from semi-quantitative Sirius Red staining (Figure 6B) and the subsequent quantification (*p* < 0.001) (Figure 6C). Similarly, apparently more mineralization nodules were detectable upon Alizarin Red staining in the presence of lithium on day 21 (Figure 6D). Spectrophotometric quantification of the extracted ARS–dye complex also confirmed significantly increased mineralization (*p* < 0.001) in MC3T3-E1 monolayer cultures in the presence of lithium as compared to the lithium-free condition (Figure 6E).

In contrast, in a 3D environment lithium at the same concentration surprisingly inhibited differentiation and mineralization of MC3T3-E1 spheroids: on day 14, Alp activity was significantly reduced (*p* < 0.001) (Figure 6F), as was type I collagen deposition and osteocalcin staining intensity on day 21 (Figure 6G). Furthermore, lithium suppressed biomineralization in spheroids cultured with the chemical for 21 days as compared with those cultured without. This was macroscopically confirmed by histochemical Alizarin Red and von Kossa staining (Figure 6G), subsequent quantification of calcium deposition (Figure 6H), and quantitative micro-CT analyses (Figure 6I–K).

Taken together, a significantly lower extracellular calcium content (*p* < 0.001) was evident in spheroids cultured in the presence of lithium compared to spheroids cultured without it (Figure 6H). Micro-CT analyses revealed that accumulation of volumes of moderately (*p* < 0.01, Figure 6J) and strongly (*p* < 0.05, Figure 6K) mineralized deposits within the spheroids were reduced by lithium. In line with this, the mean voxel intensity of the spheroids of the lithium-treated group was also significantly lower (*p* < 0.01) than that of control spheroids without addition of lithium (Figure 6I).

## 4. Discussion

Compelling evidence now suggests that 3D spheroid culture is far superior to 2D monolayer culture as non-physiological 2D conditions are hardly representative of cells residing in a complex tissue microenvironment [50,51]. With low success rates in clinical trials, drug discovery remains a slow and costly business, with more than 50% of all drugs failing in Phase II or III due to a lack of efficacy and unwanted side effects [51]. New preclinical models that better recapitulate in vivo biology and microenvironmental factors are thus urgently needed. Of note, compared to cost-intensive animal models, assays using cultured spheroids are simple, fast and cost-effective as well as versatile and easily reproducible [51].

While 3D culture models of many other organs have already been routinely used for in vitro assays, research on osteoblasts and their progenitor cells is still largely based on conventional monolayer cultures. Yet, no generally accepted spheroid model for osteoblast(-like) cells has been established and major drawbacks of reported approaches were that biomineralization was either not described [21,22,23,24] or not (reliably) quantified [25,26]. Importantly, the ECM has been shown to alter drug responses, while its biochemical composition and mechanical properties are known to steer cell behavior differently in 2D and 3D, respectively [51].

Here, we established a straightforward 3D scaffold-free spheroid culture model with well-established mammalian MC3T3-E1 cells in 96-well formats and demonstrated quantifiable sequential, and progressive, mineralization patterns. From classic osteogenic differentiation in 2D it is long known that mineralization occurs where monolayers spontaneously cluster to form spots of higher cell density, so-called “bone-like” nodules [52], leaving large remainder areas unmineralized. For MC3T3-E1 cells, these nodules were shown to consist of different cell layers [29], with central cells exhibiting morphological characteristics of young matrix-embedded osteocytes, with peripheral layers of osteoblast-like cells and a surrounding periosteum-like layer of fibrous matrix and large cells [29].

Currently, 3D culture techniques are frequently categorized into non-scaffold, anchorage-independent and scaffold-based 3D culture systems or hybrids thereof [51]. Scaffold-free models rely on cellular self-aggregation, having the advantage that they are “assumption-free”; that is, models operate without assuming that the biogel or scaffold used is either completely reproducible and not influencing the cell behavior in any way other than expected and thereby minimizing batch-to-batch variability.

In contrast to earlier reports [20], our MC3T3-E1 spheroids only showed a somewhat variable shape at the beginning of the culture period, which rapidly stabilized after a few days of in vitro maturation. Importantly, our spheroids revealed a three-dimensional morphological representation of essential characteristic of localized bone nodules in 2D culture [29]. By electron microscopy and histology, we showed that osteogenically cultured MC3T3-E1 spheroids are agglomerates of heterogeneous cells at various concentric differentiation stages: cells of the outer spheroid layer are elongated, fibroblast-like and embedded in a fibrous unmineralized matrix. Similar fibrous capsules were found in pellet culture of human primary osteoblast-like cells [23,53]. In contrast, inside MC3T3-E1 spheroid cores, cells differentiate towards mature osteoblasts secreting collagen type I and a radially expanding mineralization of the ECM at day 14. Notably, cells from this progenitor cell line thus appear to differentiate into distinct phenotypes depending on their spatial localization within the spheroid. This underscores the potential contribution of a three-dimensional environment, resulting in diffusion gradients of oxygen, nutrients, and growth factors, as opposed to conventional monolayer culture [15,51,54,55]. For MC3T3-E1 cells in 3D, earlier studies showed a hypoxia-related increase in *Vegfa* gene expression compared to monolayer culture [21]. Of note, also during endochondral ossification increased Hif-1α levels are detectable in the condensed mesenchyme [56,57,58]. Interestingly, as our Ki67 stainings and DNA measurements did not indicate proliferation, we assume that MC3T3-E1 cells rather migrated towards the core region to cause condensation, which would show parallels to embryonic osteogenesis, which also begins with cell condensation through migration of mesenchymal cells towards sites of subsequent bone formation [59]. Our results are thus in agreement with the in vivo situation, where condensation does also not result from increased proliferative activity but rather from migration. 

A reciprocal relationship between proliferation and differentiation is established in osteogenic 2D cultures, though [31,60], dividing osteogenesis into three distinct stages: (i) proliferation, (ii) extracellular matrix deposition and maturation, and (iii) mineralization. Here, proliferation is indispensable to later differentiation [61]. We demonstrated that proliferation is not a prerequisite for differentiation of MC3T3-E1 into spheroids and is in line with data showing that spheroidal culture initiates osteoblastic cell cycle arrest [21].

In vivo, mesenchymal condensation must reach a critical size to allow indispensable cell–cell and cell–ECM contacts to initiate differentiation [59,62]. This is in line with a minimal size requirement of differentiating and mineralizing MLO-A5 cell aggregates in vitro [63], and fits with the relative substantial diameter of our MC3T3-E1 spheroids. However, a risk of large-diameter spheroid culture is the formation of a necrotic core due to a lack of oxygen [55,64]; however, we have no indication of this from histology. Between day 14 and day 21, TUNEL staining confirmed an increasing number of apoptotic cells in differentiating spheroids, however, which corresponds to a decreased DNA content after day 21 under differentiation conditions. From the absence of cell death in similar-sized control spheroids, and the late time point in differentiating spheroids, we conclude that oxygen or nutrient deprivation was not a likely cause of the apoptotic cells, though. Accordingly, our spheroids have an appropriate size. Of note, mature osteoblasts also undergo apoptotic cell death in vivo [65], and a simultaneous occurrence of apoptosis in differentiating spheroids and the onset of mineralization is in support of a physiological cause. 

RUNX2, a Runt domain-containing transcription factor, is considered an early differentiation marker and a master regulator of skeletogenesis [66], with its expression emerging in pre-osteoblasts, peaking in immature osteoblasts, but decreasing in mature osteoblasts [67]. RUNX2 is essential for committing MSCs to osteoprogenitor cells [33] and for both endochondral and intramembranous ossification [66]. Under both culture conditions, on days 7 and 14, *Runx2* gene expression was increased as compared to the start, which indicates that MC3T3-E1 cells in spheroids differentiate from a pre-osteoblastic towards an immature osteoblast phenotype. This is in line with our data, showing a higher RUNX2 protein expression specifically under differentiating conditions, on day 14. Immunohistology further revealed interesting spatial differences in RUNX2 expression within one spheroid, which is a novel finding. Notable, only a thin rim between the condensed core and the outer fibrous layer in differentiating spheroids stains RUNX2 positive, which is in agreement with previous observations from intramembranous bone formation in chicken embryos [68]. Furthermore, peripheral cells are considered less differentiated and express early osteogenic markers, unlike cells in the center express later osteogenic markers, such as osteopontin [68,69],. Interestingly, also during ossification, the fibrous and the cambium layer of the periosteum are also formed peripherally to the mesenchymal condensation [70], with elongated fibroblasts in the outer region and a cell-rich inner cambium layer of mesenchymal progenitors and immature osteoblasts [71]. RUNX2 further induces the expression of a number of major bone matrix protein marker genes, including *Col1a1*, *Spp1*, and *Bglap2,* in vitro [67], which were thus briefly studied subsequently.

First, as it serves as a scaffold for the deposition of hydroxyapatite and is known to be secreted by mature osteoblasts, type I collagen expression (i.e., *Col1a1*) was screened as an indicator of progressive differentiation [72]. Within the first week of culture, compared to its starting point, *Col1a1* gene expression increased in differentiating spheroids to further increase by day 14. In contrast, no upregulation of its expression was evident in the controls. This expression pattern differs somewhat from monolayer cultures where, in primary fetal calvaria-derived osteoblasts, *Col1a1* expression is highest at the beginning of the culture, i.e., during the proliferation phase, and subsequently decreased significantly at the end of the proliferative phase with the onset of differentiation [60] even in MC3T3-E1 cells [31]. While proliferation positively correlated with type I collagen biosynthesis in 2D [61], proliferation was neglectable in spheroids and does not appear to affect *Col1a1* gene expression. However, from day 14 onwards type I collagen deposition is visible in spheroid core regions, being largely in agreement with its expression pattern in 2D [31]. Of note, another study reported a decreased *Col1a1* expression in primary murine calvaria-derived osteoblasts [45] and even in MC3T3-E1 cells in 2D [21]. Yet, these seemingly contradictory results may warrant further investigation.

Osteopontin, *Spp1*/*Bsp-1*, is considered a later stage differentiation marker [73]. During the first 14 days of MC3T3-E1 spheroid culture, *Spp1* expression did not differ between conditions; however, cores stained positive for osteopontin from day 21 onwards, and then only under differentiating culture conditions. From experiments in vivo [74] and in vitro using MC3T3-E1 cells [75], this molecule was further suggested to be an inhibitor of mineralization. However, other evidence suggests that the non-collagenous bone sialoprotein osteopontin is necessary to initiate mineralization [76]. While osteopontin may inhibit extracellular matrix mineralization through affecting the nucleation and/or growth of mineral crystals [77], osteopontin-deficient mice were reported to have an increased mineralization of trabecular bones at adult age [74]. Currently, the potential of osteopontin to alter the cell-maturation characteristics of osteoblasts is thus still a bit controversial. Such contradiction may result from a multitude of factors, like different cell types (primary rat osteoblasts compared with MC3T3-E1 pre-osteoblastics) or the method of osteopontin manipulation. Importantly, gene expression analyses of 2D-cultured primary rat calvaria-derived cells revealed a biphasic expression pattern of osteopontin, with relative much higher expression levels during the early proliferation and later mineralization phase, respectively [60,61]. This is in agreement with its biphasic expression in 2D-cultured MC3T3-E1 cells upon BMP-2 treatment [78] and our own results in 3D. Interestingly, it has been shown that loss of osteopontin does not appear to alter the expression of other major osteogenic markers, such as *Bglap* [77]. In future studies, our model may help to further clarify the still controversial role of osteopontin during bone mineralization.

Osteocalcin, encoded by the *Bglap* gene, is a vitamin K-dependent calcium-binding protein [79] which is secreted by differentiated osteoblasts before the onset of mineralization, and thus is considered a late osteogenic marker [80]. While its exact role is still unclear it may inhibit [81] or modulate bone mineral deposition on collagen fibers [82]. In spheroids under osteogenic conditions, *Bglap* expression increased significantly during the first two weeks of culture compared to controls, reaching a maximum on day 14, which indicates pre-osteoblastic differentiation. Interestingly, as compared to the starting point of the culture, its expression increased even in controls and may indicate that these spheroids also undergo some degree of initial differentiation. Noteworthy, IHC revealed an intriguing spatiotemporal expression pattern: osteocalcin expression appears first in the condensed core of differentiating spheroids at day 14, whereas it become undetectable at later time points. This was a surprise and we assume that earlier reported conformational changes upon calcium binding, and thus possible epitope camouflaging, may prevent its recognition by the used antibody [83].

Due to its importance, we next also investigated (i) expression (*Alpl*) and (ii) activity of the tissue-nonspecific alkaline phosphatase. Mineralization is the synthesis, inside matrix vesicles, of hydroxyapatite crystals that bud from the outer membrane of osteoblasts to expand into the ECM and its later accumulation between collagen fibrils [84]. High upregulation, and increased activity, of *Alpl*/ALP specifically under mineralizing culture conditions is in agreement with its activation by β-glycerophosphate [84]. Our data thus further matches *Alpl* expression profiles in osteogenically differentiated human BMSCs [85] or during human osteoblast differentiation [86].

The critical endpoint in osteoblast differentiation studies is the extent of mineralization. Quantifiable mineralization in spheroids cultured in the differentiation medium started on day 14, which is in agreement with findings from 2D cultures of this cell line [31]. Using generally accepted von Kossa and Alizarin Red staining, we evaluated the extent of mineralization semi-quantitatively [87]. Interestingly, mineralization was not homogeneously distributed throughout the entire spheroid, but rather started in the condensed core region and then expanded radially outwards, leaving just the outer fibrous capsule and a thin rim just beneath it unmineralized. This shows considerable parallels to the intramembranous ossification process in vivo, where—following mesenchymal condensation—progenitor cells differentiate into mature osteoid-depositing osteoblasts to then mineralize the ECM through hydroxyapatite incorporation [62]. In essence, both techniques independently confirmed a progressively increasing mineralization over culture time in the osteogenically cultured spheroids only. However, despite their popularity due to their ease of use, none of these two gold standard techniques is able to detect truly hydroxyapatite-like mineral depositions in 2D, and specifically not in MC3T3-E1 cells [38]. While von Kossa stains “dystrophic mineralization of unknown origin”, Alizarin Red staining is prone to false-positive results in the presence of large amounts of calcium-binding proteins and proteoglycans [38]. We therefore validated our histochemical staining by EDX and FTIR analyses as suggested earlier by Bonewald et al. [38]. Noteworthy, in MC3T3-E1 spheroids, was that EDX confirmed the presence of calcium and phosphate in mineralized regions of the differentiating spheroids, while FTIR analyses of these spheroids confirmed an absorption spectrum resembling that of native murine bone. This is consistent with previous observations showing that MC3T3-E1 cells are ideally suited to study biomineralization in vitro as they can form mineralized matrix containing apatitic carbonates and phosphates very similar to real bone [88]. Importantly, the apatite-containing matrix formation highlights the unique potential of our spheroid culture as an in vitro model for biomimetic mineralization. To our mind, this also warrants including EDX- and FTIR-based characterization more frequently in future studies to verify hydroxyapatite-like mineral deposition. 

Finally, we used micro-computed tomography (micro-CT) as a noninvasive means to volumetrically assess and objectively quantify changes in mineral densities [39,63,89] while, in contrast, immunohistochemical staining of 3D constructs only allows a potentially biased qualitative evaluation of the mineralization process. Interestingly, in immunohistochemistry even small calcium deposits resulted in an intense and assumingly uniform mineralization of e.g., the spheroid core region. In contrast, micro-CT returned a highly diverse distribution of signal densities over the mineralized regions, being indicative of different mineralization intensities and demonstrating a much finer resolution. The strongest mineralization was found in a ring-shaped area at the outer rim of the mineralized core. Strikingly, towards the end of culture, trabecular-like structures appear, which, to our knowledge, have never been shown earlier in vitro. The distribution of mineralization in our spheroids thus visually resembles structures of real bone with compacta and spongiosa. Of note, in our study subtle mineralization was evident by immunohistochemistry prior to detection by micro-CT as thresholding cut-off values were chosen conservatively to not overestimate mineralization. Despite the enormous advantages of micro-CT analyses, proper thresholding is challenging and a matter of experience and should always be compared to immunohistochemistry. In our case, however, both methods largely confirm each other.

Recently, a large cohort study demonstrated that lithium can significantly reduce the risk of osteoporosis [90], which confirms earlier results from animal studies, demonstrating a positive effect on (osteoporotic) bone regeneration in humans [91,92]. Ultimately, we thus used lithium chloride as a well-established inhibitor of cWnt signaling, which has been reported to stimulate or inhibit osteoblast differentiation and mineralization in vitro [86], to study its potentially different pharmacological effects on MC3T3-E1 in 2D and 3D. Many cell types respond in significantly different fashion to drugs depending on their cell culture context, i.e., whether they are cultured as conventional monolayers or in 3D [15,93]. We have now demonstrated that the effect of glycogen synthase kinase 3β (GSK-3β)-inhibitor lithium on the differentiation and mineralization of MC3T3-E1 was fundamentally different between 2D and 3D cultures. While 5 mM LiCl increased differentiation and mineralization in 2D-cultured MC3T3-E1 cells, we observed a significant inhibiting effect in spheroids. Other authors also showed that lithium concentrations of 3 mM and 7 mM, respectively, increased alkaline phosphatase activity and mineralization [49]. Lithium directly inhibits GSK-3β mediated degradation of β-catenin via competition with magnesium ions [94] and thus leading to Lrp5-independent activation of the canonical Wnt signaling pathway [11]. Wnt signaling is complexly regulated, with different effects on osteogenic differentiation during distinct stages. It has been shown for murine pre-osteoblast cell line KS483 that partial downregulation of cWnt signaling is required for terminal differentiation and matrix mineralization [95]. Increased expression of Wnt antagonists was also observed in MC3T3-E1 cells during the late differentiation phase, suggesting that a negative feedback loop is required for terminal osteoblast maturation [96]. Thus, the highly regulated time- and dosage-dependent cWnt signaling activity is critical for osteoblast maturation and mineralization. It is well-accepted that endogenous cWnt signaling activity differs depending on whether mesenchymal stem cells (MSC) are cultured two-dimensionally or spheroidally. Of note, during osteogenic differentiation of 2D-cultured MSC, only low (or even no) endogenous Wnt activity is detectable [97,98]. Also, for 2D-cultured MC3T3-E1 cells, microarray analyses revealed low basal expression of cWnt signaling components [96]. Thus, during 2D osteogenic differentiation of MSCs, cWnt signaling is apparently not required, which is in strong contrast to findings in vivo. Additionally, 2D in vitro studies on the pro-osteogenic effect of lithium required doses up to 10-fold higher than in vivo, indicating that 2D culture of osteogenic cells may result in a non-physiological regulation of the cWnt signaling pathway. Interestingly, high cWnt activity is detectable in MSC spheroid cultures [97,98], where cWnt signaling activity was remarkably detected mainly in central spheroid regions [98], which is in agreement with our findings. Thus, due to the perhaps endogenously increased cWnt activity in spheroids, the lithium concentration of 5 mM might be too high, resulting in an insufficient downregulation of cWnt signaling and therefore a terminal differentiation block in 3D culture. Our spheroid model could thus contribute to new insights into the temporal and spatial regulation of cWnt signaling, as spheroid culture may lead to a more physiological cWnt signaling activity during osteogenic differentiation.

In summary, we established a scaffold-free 3D biomimetic mineralization model based on well-established MC3T3-E1 osteoprogenitor cells. Our model system paves the way for studying physiological biomineralization in vitro and performing high-throughput drug screenings.

## 5. Conclusions

We developed a murine osteoblast progenitor cell-based spheroidal culture model that mimics physiological mineralization during in vitro osteogenesis. The model is based on commercially available resources, inexpensive, not reliant on unnecessary additional biomaterials, robust and high-throughput compatible. Expression profiles of characteristic differentiation stage-dependent osteogenic marker genes were confirmed and validated by corresponding immunohistochemical analyses. A centrally starting and radially expanding intense mineralization pattern was evident from gold standard histochemistry and objectively quantified by micro-CT analyses, which non-invasively revealed a biomineralization process with a dynamically increasing mineral density over a culture period of 28 days. SEM-EDX and FT-IR ultimately confirmed a native bone-like hydroxyapatite mineral deposition with trabecular-like bone structures ex vivo. We believe our model holds a lot of potential to improve future pharmacological screenings of anabolic drugs aiming to improve bone strength.

## Figures and Tables

**Table 1:** RT-qPCR primer information. Table shows official NCBI gene symbols, accession numbers, nucleotide primer sequence, annealing temperature, and verified amplicon length (in bp).

Gene	Primer Sequence 5″ → 3″	Annealing Temp [°C]	Amplicon Length	Accession No.
Alpl	F: CCA ACT CTT TTG TGC CAG AGA R: GGC TAC ATT GGT GTT GAG CTT TT	60.0	110 mer	NM_001287172.1
Bglap	F: GCC CAG ACC TAG CAG ACA C R: TGG GCT TGG CAT CTG TGA G	59.0	97 mer	NM_007541
Col1a1	F: CTA CTA CCG GGC CGA TGA TG R: CGA TCC AGT ACT CTC CGC TC	59.0	188 mer	NM_007742.3
Hprt	F: TCA GTC AAC GGG GGA CAT AAA R: GGG GCT GTA CTG CTT AAC CAG	61.0	142 mer	NM_013556.2
Runx2	F: GCC AGG CAG GTG CTT CAG AAC T R: CTG GGC GGG GTG TAG GTA AAG	59.0	133 mer	NM_001146038.2
Sdha	F: GGA ACA CTC CAA AAA CAG ACC T R: CCA CCA CTG CGT ATT GAG TAG AA	60.0	106 mer	NM_023281.1
Spp1	F: GAG AAG CTT TAC AGC CTG CAC C R: ATT GGA ATT GCT TGG AAG AGT TTC T	59.0	150 mer	NM_001204233.1

## Data Availability

Original data supporting findings of this study are available upon request from the corresponding authors.
